# Comparative genomics of proteins involved in RNA nucleocytoplasmic export

**DOI:** 10.1186/1471-2148-11-7

**Published:** 2011-01-11

**Authors:** Mariana Serpeloni, Newton M Vidal, Samuel Goldenberg, Andréa R Ávila, Federico G Hoffmann

**Affiliations:** 1 Departamento de Biologia Celular e Molecular, Universidade Federal do Paraná - UFPR, Curitiba, Brazil; 2 Instituto Carlos Chagas - ICC, Curitiba, Brazil; 3 School of Biological Sciences, University of Nebraska, Lincoln, USA; 4 Department of Biochemistry and Molecular Biology, Mississippi State University, Mississippi State, USA

## Abstract

**Background:**

The establishment of the nuclear membrane resulted in the physical separation of transcription and translation, and presented early eukaryotes with a formidable challenge: how to shuttle RNA from the nucleus to the locus of protein synthesis. In prokaryotes, mRNA is translated as it is being synthesized, whereas in eukaryotes mRNA is synthesized and processed in the nucleus, and it is then exported to the cytoplasm. In metazoa and fungi, the different RNA species are exported from the nucleus by specialized pathways. For example, tRNA is exported by exportin-t in a RanGTP-dependent fashion. By contrast, mRNAs are associated to ribonucleoproteins (RNPs) and exported by an essential shuttling complex (TAP-p15 in human, Mex67-mtr2 in yeast) that transports them through the nuclear pore. The different RNA export pathways appear to be well conserved among members of Opisthokonta, the eukaryotic supergroup that includes Fungi and Metazoa. However, it is not known whether RNA export in the other eukaryotic supergroups follows the same export routes as in opisthokonts.

**Methods:**

Our objective was to reconstruct the evolutionary history of the different RNA export pathways across eukaryotes. To do so, we screened an array of eukaryotic genomes for the presence of homologs of the proteins involved in RNA export in Metazoa and Fungi, using human and yeast proteins as queries.

**Results:**

Our genomic comparisons indicate that the basic components of the RanGTP-dependent RNA pathways are conserved across eukaryotes, and thus we infer that these are traceable to the last eukaryotic common ancestor (LECA). On the other hand, several of the proteins involved in RanGTP-independent mRNA export pathways are less conserved, which would suggest that they represent innovations that appeared later in the evolution of eukaryotes.

**Conclusions:**

Our analyses suggest that the LECA possessed the basic components of the different RNA export mechanisms found today in opisthokonts, and that these mechanisms became more specialized throughout eukaryotic evolution.

## Background

Protein synthesis in all living cells involves the transcription of DNA into messenger RNA (mRNA) and its subsequent translation into polypeptides. In prokaryotes, transcription and translation are physically and temporally linked, and each mRNA molecule is translated by the ribosomes as it is transcribed. By contrast, in eukaryotes transcription and mRNA processing are physically and temporally separated from translation by the nuclear membrane. This separation is hypothesized to have been a major factor in the emergence of the nuclear membrane [1]. As a result of the establishment of the nuclear membrane, the different RNA species involved in protein synthesis such as mRNAs, ribosomal RNAs (rRNAs), and transfer RNAs (tRNAs), need to be shuttled from the nucleus to the cytoplasm. The general model of RNA export involves exportins as transport receptors that carry RNA through the nuclear pore complex (NPC) in a RanGTP-dependent manner [2]. In Metazoa and Fungi, the nuclear export of most RNA species, such as microRNAs (miRNAs), rRNAs, small nuclear RNAs (snRNAs), and tRNAs, has been shown to follow the RanGTP-exportin model of transport, and specific exportins are involved with the different export pathways. However, nuclear export of most mRNAs does not follow the RanGTP-exportin pathway [3-5]. The mRNA export machinery is highly integrated with mRNA processing, and it includes a different set of nuclear transport adaptors plus other mRNA binding proteins, RNA helicases, and NPC associated proteins [4-6].

It is not known whether the proteins involved in the different RNA export pathways are conserved among the different eukaryotic supergroups, and which components of the nucleocytoplasmic export of RNA are traceable to the last eukaryotic common ancestor (LECA). The goal of this study was to explore the evolutionary history of the different RNA export pathways across a diverse array of eukaryotes, with special emphasis on members of the supergroups Excavata and Chromalveolata, in order to identify lineage-specific innovations and make inferences regarding RNA export evolution. To do so, we screened the genome of 65 species of eukaryotes to explore the presence of homologs of proteins involved in RNA export in Metazoa and Fungi. Results from our bioinformatic comparisons suggest that the basic components of the RanGTP-dependent RNA pathways are conserved across eukaryotes, whereas proteins involved in RanGTP-independent mRNA export are less conserved.

## Methods

### Data Sources

We identified sixty four proteins from baker's yeast (*S. cerevisiae) *experimentally shown to be involved in RNA export and their putative human orthologs from Ensembl (release 54), plus PHAX, a human protein involved in RNA export that apparently lacks a yeast ortholog. For simplicity, proteins have been named according to their human ortholog when present, the full list of proteins used is provided in Table S1 (Additional File 1). Using all the proteins previously identified to seed bionformatic searches, we interrogated the genomes of 65 eukaryotic species to identify their putative homologs. Our initial set of species included representatives of five of the different eukaryotic supergroups (Opisthokonta, Amoebozoa, Chromalveolata, Plantae, and Excavata). The genomes of most species were downloaded from the NCBI Reference Sequence (RefSeq) collection except where noted (see Table 1). The initial set included 2 or more representatives of the genera *Caenorhabditis*, *Entamoeba*, *Cryptosporidium*, *Leishmania*, *Plasmodium*, and *Trypanosoma *which provided taxonomic redundancy and served as consistency controls for our protocols.

**Table 1:** List of species included in this study

*Species*	Code	Lineage	Supergroup	Data Source
*Homo sapiens*	Hsa	Metazoa	Opisthokonta	NCBI RefSeq
*Mus musculus^a^*	Mmu	Metazoa	Opisthokonta	NCBI RefSeq
*Rattus norvegicus^a^*	Rno	Metazoa	Opisthokonta	NCBI RefSeq
*Gallus gallus^a^*	Gga	Metazoa	Opisthokonta	NCBI RefSeq
*Xenopus tropicalis^a^*	Xtr	Metazoa	Opisthokonta	NCBI RefSeq
*Danio rerio^a^*	Dre	Metazoa	Opisthokonta	NCBI RefSeq
*Takifugu rubripes*	Fru	Metazoa	Opisthokonta	Fugu Genome Project
*Ciona intestinalis*	Cin	Metazoa	Opisthokonta	NCBI RefSeq
*Anopheles gambiae^a^*	Aga	Metazoa	Opisthokonta	NCBI RefSeq
*Drosophila melanogaster*	Dme	Metazoa	Opisthokonta	NCBI RefSeq
*Apis mellifera^a^*	Ame	Metazoa	Opisthokonta	NCBI RefSeq
*Caenorhabditis briggsae ^a^*	Cbr	Metazoa	Opisthokonta	NCBI RefSeq
*Caenorhabditis elegans*	Cel	Metazoa	Opisthokonta	NCBI RefSeq
*Monosiga brevicollis*	Mbr	Choanoflagellate	Opisthokonta	NCBI RefSeq
*Saccharomyces cerevisiae^a^*	Sce	Fungi	Opisthokonta	NCBI RefSeq
*Candida glabrata*	Cgl	Fungi	Opisthokonta	NCBI RefSeq
*Ashbya gossypii^a^*	Ago	Fungi	Opisthokonta	NCBI RefSeq
*Kluyveromyces lactis*	Kla	Fungi	Opisthokonta	NCBI RefSeq
*Debaryomyces hansenii*	Dha	Fungi	Opisthokonta	NCBI RefSeq
*Yarrowia lipolytica*	Yli	Fungi	Opisthokonta	NCBI RefSeq
*Magnaporthe oryzae^a^*	Mor	Fungi	Opisthokonta	NCBI RefSeq
*Neurospora crassa^a^*	Ncr	Fungi	Opisthokonta	NCBI RefSeq
*Fusarium oxysporum^a^*	Fox	Fungi	Opisthokonta	NCBI RefSeq
*Aspergillus fumigatus*	Afu	Fungi	Opisthokonta	NCBI RefSeq
*Schizosaccharomyces pombe*	Spo	Fungi	Opisthokonta	NCBI RefSeq
*Cryptococcus neoformans*	Cne	Fungi	Opisthokonta	NCBI RefSeq
*Ustilago maydis^a^*	Uma	Fungi	Opisthokonta	NCBI RefSeq
*Encephalitozoon cuniculi*	Ecu	Fungi	Opisthokonta	NCBI RefSeq
*Dictyostelium discoideum*	Ddi	Mycetozoa	Amoebozoa	NCBI RefSeq
*Entamoeba dispar ^a^*	Edi	Archamoebae	Amoebozoa	NCBI RefSeq
*Entamoeba histolytica*	Ehi	Archamoebae	Amoebozoa	NCBI RefSeq
*Arabidopsis thaliana*	Ath	Streptophyta	Plantae	NCBI RefSeq
*Oryza sativa*	Osa	Streptophyta	Plantae	NCBI RefSeq
*Populus trichocarpa*	Pop	Streptophyta	Plantae	NCBI RefSeq
*Physcomitrella patens*	Ppa	Streptophyta	Plantae	NCBI RefSeq
*Chlamydomonas reinhardtii*	Cre	Chlorophyta	Plantae	NCBI RefSeq
*Volvox carteri*	Vca	Chlorophyta	Plantae	NCBI RefSeq
*Ostreococcus lucimarinus^a^*	Olu	Chlorophyta	Plantae	NCBI RefSeq
*Cyanidioschyzon merolae*	Cme	Rhodophyta	Plantae	U. of Tokyo
*Guillardia theta^b^*	Gth	Cryptophyta	Chromalveolata	NCBI RefSeq
*Bigelowiella natans^b^*	Bna	Cercozoa	Rhizaria	NCBI RefSeq
*Emiliania huxleyi^c^*	Ehu	Haptophyta	?	Joint Genome Institute
*Theileria parva*	Tpa	Alveolata	Chromalveolata	NCBI RefSeq
*Plasmodium berghei ^a^*	Pbe	Alveolata	Chromalveolata	NCBI RefSeq
*Plasmodium falciparum*	Pfa	Alveolata	Chromalveolata	NCBI RefSeq
*Plasmodium knowlesi ^a^*	Pkn	Alveolata	Chromalveolata	NCBI RefSeq
*Plasmodium vivax ^a^*	Pvi	Alveolata	Chromalveolata	NCBI RefSeq
*Plasmodium yoelii ^a^*	Pyo	Alveolata	Chromalveolata	NCBI RefSeq
*Toxoplasma gondii*	Tgo	Alveolata	Chromalveolata	ToxoDB
*Cryptosporidium hominis ^a^*	Cho	Alveolata	Chromalveolata	NCBI RefSeq
*Cryptosporidium parvum*	Cpa	Alveolata	Chromalveolata	NCBI RefSeq
*Paramecium tetraurelia*	Pte	Alveolata	Chromalveolata	NCBI RefSeq
*Tetrahymena thermophila*	Tth	Alveolata	Chromalveolata	NCBI RefSeq
*Phaeodactylum tricornutum*	Ptr	Stramenopila	Chromalveolata	NCBI RefSeq
*Thalassiosira pseudonana*	Tps	Stramenopila	Chromalveolata	NCBI RefSeq
*Phytophthora infestans*	Pin	Stramenopila	Chromalveolata	NCBI RefSeq
*Leishmania braziliensis ^a^*	Lbr	Kinetoplastida	Excavata	NCBI RefSeq
*Leishmania infantum ^a^*	Lin	Kinetoplastida	Excavata	NCBI RefSeq
*Leishmania major*	Lma	Kinetoplastida	Excavata	NCBI RefSeq
*Trypanosoma brucei*	Tbr	Kinetoplastida	Excavata	NCBI RefSeq
*Trypanosoma cruzi ^a^*	Tcr	Kinetoplastida	Excavata	NCBI RefSeq
*Trypanosoma vivax ^a^*	Tvi	Kinetoplastida	Excavata	GeneDB
*Naegleria gruberi*	Ngr	Heterolobosea	Excavata	NCBI RefSeq
*Giardia lamblia*	Gla	Diplomonadida	Excavata	NCBI RefSeq
*Trichomonas vaginalis*	Tva	Parabasalia	Excavata	NCBI RefSeq

*^a ^*Results for these species are available in the Supplementary Online Material.

*^b ^*The nucleomorph genomes of these species were included in the searches. Despite the phylogenetic position of the hosts, they were members of placed close to Plantae in the tables because of the algal origin of the nucleomorphs.

*^c ^*The phylogenetic position of *Emiliana *is still unclear (see [11,12]).

### Bioinformatic searches

Bioinformatic searches were locally performed using the BLASTP, PSI-BLAST and TBLASTN algorithms [7], part of the NCBI C++ toolkit [8]. In the case of BLASTP, we compared results obtained under three different matrices (BLOSUM62, BLOSUM45 and PAM250) and in the case of PSI-BLAST we compared the results obtained for 2, 3 and 5 iterations. There were no major differences between the different search strategies implemented. Thus, we report results for BLASTP with the BLOSUM62 matrix in the manuscript. All results, including Reciprocal Best Hit (RBH), also evaluated using BLASTP with the BLOSUM62 matrix, are presented on Tables S2 and S3 (Additional File 1). Results were then ranked into 5 categories (1-5; with 1 being the most conserved) as described below. Hits with E-values better than 10^-5 ^were classified according to the following criteria: category 1 [strong similarity], a candidate homolog sequence (CHS) of similar length to the query protein sequence (QS), showing a similarity of 60% or greater, and having a match that covers at least 80% of the QS; category 2 [similar], a CHS of similar length to the QS, similarity higher than 50%, and covering at least 60% of QS; category 3 [weak similarity], a CHS of similar length to QS, with similarity higher than 40%, and covering at least 45% of QS; category 4 [partial similarity], a CHS having 30% similarity or greater, and covering at least 30% of QS; and category 5 [very weak similarity], a CHS with E-value better than 10^-5 ^that did not match any of the criteria above; candidates with E-value worse than 10^-5 ^were not classified (NC). In cases where human and yeast orthologs were present, results from the two queries were assessed together to ensure consistency. Proteins in categories 1 and 2 were considered as strong homology candidates to the query sequences, proteins in category 3 were considered as potential homologs of the query sequences, proteins in categories 4 and 5 were considered as showing some local homologies due to sharing conserved domains. Exemplary alignments for each category are provided in Additional File 2. TBLASTN only improved results by uncovering category 5 CHS in cases where no CHS had been identified, and the results are presented in Table S3 (Additional File 1) but are not discussed further. It is important to note that a negative result does not prove the absence of a particular protein homolog, it is just an indication that our protocols, based on sequence similarity, were not able to identify a homolog candidate.

### Reconstruction of RNA export pathways in LECA

**Figure 1:**
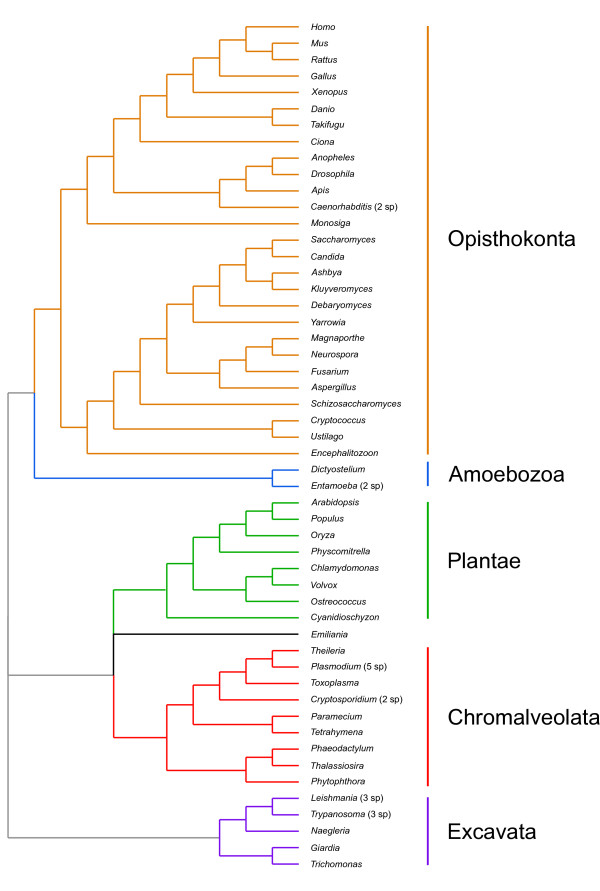
**Phylogenetic relationships among the species included in our study, based on results from four recent studies that group the six supergroups into three different megagroups **[9-12]. Relationships within Fungi follow Fitzpatrick et al. [73]. The genomes of the nucleomorphs of *Guillardia theta *and *Bigelowiella natans *were included in the bioinformatic searches but not in tree because its phylogenetic position does not match the position of the host. The different eukaryotic supergroups are highlighted. The phylogenetic position of *Emiliana *is still unclear (see [11,12]), and thus, it was left in black.

Our reconstruction of the different RNA export pathways in the LECA was based on a tree adapted from four recent reports that group the six eukaryotic supergroups into three separate megagroups [9-12]. The first megagroup includes Opisthokonta and Amoebozoa, the second one includes Chromalveolata, Plantae, and Rhizaria, and the third one corresponds to Excavata (Figure 1). Despite significant advances in our understanding of eukaryotic phylogeny, no consensus has emerged regarding the placement of the root of the eukaryotic tree (see [12-14] for a discussion). To err on the side of caution, we have based our inferences on an unrooted tree (Figure 1), and set relatively stringent criteria in order to reconstruct the different RNA export pathways in the LECA. Proteins inferred as likely to be present in the LECA had to have matches in categories 1 or 2 in members of the three megagroups. For example, a protein with strong homology matches in Opisthokonta or Amoebozoa, strong homology matches in Chromalveolata or Plantae, and strong homology matches in Excavata would be considered as likely to have already been present in the LECA. In turn, proteins with matches in the top 3 categories in members of the three megagroups were inferred as probably present in the LECA.

Three additional sets of proteins were included for validation purposes. Firstly, we included members of the SAGA transcription complex (Table S2 and S3, Additional File 1), which is specific of fungi lineages [15,16]. As an additional test, we included the proteins that make the U5snRNP spliceosomal complex as reported by Collins and Penny [17] and checked that our results were consistent with those reported previously (Table S4, Additional File 1). Finally, we compared our inferences with those of two previous studies that focused on the components of the NPC [18,19], and found that results are similar for all overlapping proteins, suggesting that the search protocols are comparable (Table S5, Additional File 1). Importantly, our analysis broadened the number of proteins analyzed and cover a much wider portion of eukaryotic diversity.

## Results and discussion

**Figure 2:**
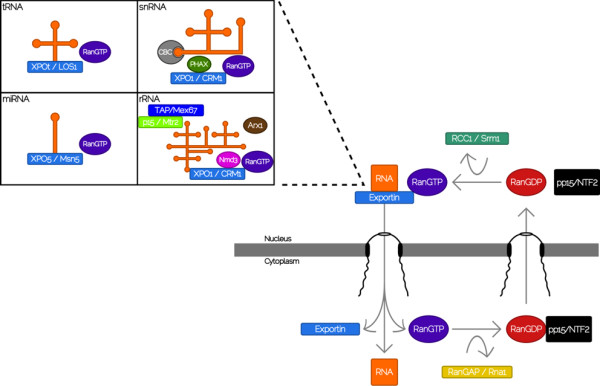
**Schematic representation of the RanGTP-dependent RNA export pathways in yeast (see **[4]**for a full description)**. Nuclear export of miRNA, rRNA, snRNA, and tRNA has been shown to follow the RanGTP-dependent exportin model. The nuclear export receptors, exportins, bind nuclear cargo together with RanGTP in the nucleus to form a ternary complex (RNA-exportin-RanGTP) that is translocated to the cytoplasm. The complex dissociates in the cytoplasm upon hydrolysis of RanGTP to release the cargo molecule. Different exportins are preferentially involved with the different RNA export pathways. Exportin t (XPOt) is involved with tRNA export, Exportin 5 (XPO5) in miRNA export whereas Exportin 1 (XPO1) is in charge of snRNA and rRNA export, as shown in the corresponding box. In the case of snRNA and rRNA export, additional adaptors are needed. After GTP hydrolysis in the cytoplasm, the import receptor, NTF2, carries RanGDP into the nucleus, where nucleotide exchange occurs by RCC1 to generate RanGTP.

**Figure 3:**
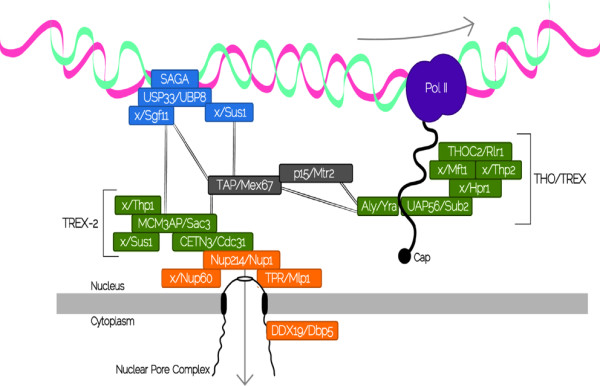
**Schematic representation of the mRNA export pathway in yeast (see **[4]**for a full description)**. mRNAs associate with protein factors into messenger RNPs (mRNPs) which are then exported through the NPC by the TAP/p15 heterodimer (Mex67/Mtr2 in yeast), that functions as a general mRNA export receptor. TAP/p15 operates in association with TREX (Transcription/Export), a complex that connects transcription with mRNA export and the THO complex. In yeast there is a an additional complex named TREX-2, that is also capable of mediating the nuclear export of mRNPs. Each box represents a human/yeast protein and the absence of human predicted orthologs, is represented by "X". Proteins are colored according to the stage in which they act and lines represent interactions between the complexes. TREX: transcription-coupled to export complex. Pol II: RNA Polymerase II.

In this study, we examined conservation of the proteins involved in RNA export across eukaryotes. The different RNA export pathways can be divided into two different groups. In opisthokonts, miRNAs, rRNAs, snRNAs, and tRNAs are exported through RanGTP-dependent pathways (Figure 2), whereas mRNA export follows a RanGTP-independent pathway (Figure 3). We examined conservation of the proteins involved in the different RNA export pathways across eukaryotes, with special focus on distinguishing components that are traceable to the LECA from those that derive from lineage-specific innovations. To facilitate the discussion, the different proteins were grouped according to the pathways they are involved with. In the case of proteins that are involved in more than one pathway, they were discussed in the context where their role appears to be better understood. Thus, results for the TAP-p15 heterodimer are discussed in the context of mRNA export, although TAP-p15 also plays a role in rRNA export. We present results for the most representative proteins in Tables 2 and 3, and complete results for all species are available in Tables S2 and S3 (Additional File 1).

**Table 2:** Bioinformatic results for proteins involved in the RanGTP-dependent RNA export pathways

		Opisthokonta										Amo		Plantae							Chromalveolata										Excavata				
					
	Queries	Hsa	Cin	Dme	Cel	Mbr	Cgl	Afu	Spo	Cne	Ecu	Ddi	Ehi	Ath	Osa	Pop	Ppa	Cre	Vca	Cme	Ehu	Tpa	Pfa	Tgo	Cpa	Pte	Tth	Ptr	Tps	Pin	Lma	Tbr	Ngr	Gla	Tva
	**Hsa_RAN**	1	1	1	1	1	1	1	1	1	1	1	1	1	1	1	1	1	1	1	1	1	1	1	1	1	1	1	1	1	1	1	1	1	1
	**Sce_Gsp1p**	1	1	1	1	1	1	1	1	1	1	1	1	1	1	1	1	1	1	1	1	1	1	1	1	1	1	1	1	1	1	1	1	1	1
	
	**Hsa_Ranbp1**	1	2	4	4	2	2	2	2	2	2	2	2	1	1	1	2	4	2	4	2	4	4	2	2	2	2	2	2	1	3	2	2	2	2
	**Sce_Yrb1p**	2	2	4	4	2	1	1	1	2	2	2	2	2	2	2	2	4	2	4	2	4	4	1	2	2	2	2	2	2	3	2	2	3	2
**Ran cycle proteins**																																			
	**Hsa_RCC1**	1	1	4	4	4	3	3	3	3	3	4	4	3	3	2	3	3	4	2	4	4	3	4	4	4	4	4	3	2	4	4	4	NC	4
	**Sce_Srm1p**	3	3	3	3	3	1	1	2	2	3	3	NC	3	3	3	3	3	3	4	3	4	4	4	4	3	4	4	3	3	4	4	3	NC	4
	
	**Hsa_RANGAP**	1	1	2	4	3	4	3	4	4	4	3	NC	3	3	3	3	4	4	3	4	NC	NC	NC	NC	4	4	3	4	4	3	4	3	4	3
	**Sce_Rna1**	4	4	4	4	4	1	1	1	2	3	4	NC	4	4	4	4	NC	3	NC	4	NC	NC	NC	NC	3	3	NC	NC	4	NC	NC	NC	NC	NC

	**Hsa_XPO5**	1	3	2	5	3	3	3	3	5	5	3	NC	3	3	5	3	5	5	5	5	5	5	5	5	NC	NC	5	5	3	5	5	3	NC	5
	**Sce_Msn5p**	3	5	NC	NC	3	1	3	3	NC	NC	3	NC	NC	NC	NC	4	NC	NC	NC	NC	NC	NC	NC	NC	NC	NC	NC	NC	3	NC	NC	4	NC	NC
	
**Exportins**	**Hsa_Xpot**	1	1	4	3	3	2	3	2	3	NC	2	5	2	3	2	2	NC	NC	3	4	NC	4	NC	5	NC	NC	3	3	3	3	3	4	5	5
	**Sce_Los1p**	3	3	NC	3	4	1	3	3	3	NC	3	5	3	4	3	3	NC	NC	NC	NC	NC	NC	NC	5	NC	NC	NC	NC	3	5	4	4	NC	NC
	
	**Hsa_XPO1**	1	1	1	1	1	1	1	1	1	2	1	4	1	1	1	1	1	1	1	1	2	2	2	1	4	2	1	1	1	2	2	1	4	3
	**Sce_Crm1p**	1	1	1	1	1	1	1	1	1	2	1	4	1	1	1	1	1	1	1	2	2	2	2	2	4	2	1	1	1	3	2	1	4	3

	**Hsa_EEFIA**	1	1	1	1	1	1	1	1	1	2	1	1	1	1	1	1	1	1	1	4	1	1	1	1	1	1	1	1	1	1	1	1	1	1
	**Sce_Tef2p**	1	1	1	1	1	1	1	1	1	2	1	1	1	1	1	1	1	1	1	4	1	1	1	1	1	1	1	1	1	1	1	1	1	1
	
	**Hsa_CBP20**	1	1	1	1	4	4	2	1	2	2	4	2	4	4	4	2	1	4	1	4	4	4	4	4	1	4	2	2	4	1	2	4	NC	1
	**Sce_Cbc2p**	3	3	3	2	5	1	2	2	3	3	3	3	2	2	2	4	3	4	4	2	2	3	4	3	3	3	4	4	4	2	1	4	NC	2
	
	**Hsa_CBP80**	1	1	1	2	NC	3	3	3	3	NC	2	NC	2	2	2	2	4	4	4	3	NC	NC	4	NC	3	NC	NC	NC	3	NC	NC	3	NC	NC
	**Sce_Sto1p**	4	5	3	3	NC	1	3	4	3	NC	4	NC	4	4	4	4	5	5	5	NC	NC	NC	NC	NC	NC	NC	NC	NC	4	NC	NC	NC	NC	NC
	
	**Hsa_PHAX**	1	3	2	4	NC	NC	NC	NC	NC	NC	NC	NC	NC	NC	NC	NC	NC	NC	NC	5	5	NC	5	5	NC	NC	NC	NC	5	NC	NC	NC	NC	NC
	**Sce-no ortholog**																																		
**Additional export factors**																																			
	**Hsa_NMD3**	1	1	1	1	1	1	1	1	1	2	1	2	1	1	1	1	4	1	1	1	2	4	4	1	1	1	1	1	1	1	1	1	2	2
	**Sce_Nmd3p**	1	1	1	1	1	1	1	1	1	2	1	2	1	1	1	1	4	1	1	1	2	4	4	1	1	1	1	1	1	1	1	1	2	2
	
	**Hsa_ZNF622**	1	1	2	3	2	3	2	2	2	NC	2	2	2	3	2	2	4	3	3	4	4	3	4	4	4	2	3	3	2	3	4	NC	3	4
	**Sce_Rei1p**	3	2	2	2	2	1	3	2	2	NC	2	3	2	2	3	2	3	4	3	4	3	5	5	3	3	2	2	4	2	3	3	NC	2	3
	
	**Hsa-no ortholog**																																		
	**Sce_Arx1p**	4	4	4	4	4	1	4	3	NC	NC	4	4	4	4	4	4	5	4	4	4	4	4	4	4	4	5	4	4	4	4	4	4	NC	NC
	
	**Hsa-no ortholog**																																		
	**Sce_Alb1p**	NC	NC	NC	NC	NC	1	NC	NC	NC	NC	NC	NC	NC	NC	NC	NC	NC	NC	NC	NC	NC	NC	NC	NC	NC	NC	NC	NC	NC	NC	NC	NC	NC	NC

Results were ranked into 5 categories (1-5; with 1 being the most conserved). NC (Not Classified). We present results for a representative subset of species in here, and full results are presented in Table S2 (Additional File 1)

**Table 3:** Bioinformatic results for proteins involved the RanGTP-independent mRNA export pathway

			Opisthokonta										Amo		Plantae							Chromalveolata										Excavata				
		Queries	Hsa	Cin	Dme	Cel	Mbr	Cgl	Afu	Spo	Cne	Ecu	Ddi	Ehi	Ath	Osa	Pop	Ppa	Cre	Vca	Cme	Ehu	Tpa	Pfa	Tgo	Cpa	Pte	Tth	Ptr	Tps	Pin	Lma	Tbr	Ngr	Gla	Tva
		**Hsa_CETN3**	1	1	4	1	1	1	4	1	1	2	1	1	1	1	1	1	1	1	2	1	1	1	1	1	1	1	1	1	1	1	1	1	1	1
		**Sce_Cdc31p**	1	1	4	1	1	1	4	1	1	2	1	2	1	1	1	1	1	1	2	1	1	1	1	1	1	1	1	1	1	1	1	1	1	1
		
		**Hsa_MCM3AP**	1	3	5	5	5	5	5	5	5	5	5	5	4	5	5	5	5	5	5	5	NC	NC	5	5	5	5	5	5	5	NC	NC	5	NC	5
		**Sce_Sac3p**	4	5	5	5	5	1	5	4	5	5	5	NC	5	5	5	5	5	5	5	5	NC	NC	NC	NC	NC	NC	5	5	5	NC	NC	4	NC	5
**TREX-2**																																				
		**Hsa-no ortholog**																																		
		**Sce_Thp1p**	4	4	3	3	3	1	NC	3	NC	NC	3	NC	3	4	NC	4	NC	NC	3	NC	NC	NC	NC	NC	3	NC	4	4	4	NC	NC	4	NC	NC
		
		
		**Hsa-no ortholog**																																		
		**Sce_Sus1p**	2	1	2	NC	2	NC	NC	2	2	NC	2	NC	NC	NC	2	2	3	NC	NC	NC	NC	NC	NC	NC	NC	NC	NC	NC	NC	NC	NC	NC	NC	NC

		**Hsa_THOC2**	1	1	1	2	4	3	4	2	4	NC	4	5	2	2	2	2	NC	4	5	4	4	5	4	NC	3	3	3	3	2	NC	NC	3	NC	NC
		**Sce_Rlr1p**	3	3	3	3	4	1	4	3	3	NC	4	5	3	3	4	3	NC	4	NC	NC	NC	5	NC	NC	4	4	4	4	4	NC	NC	4	NC	4
		
		**Hsa-no ortholog**																																		
		**Sce_Tho1p**	NC	NC	NC	NC	NC	2	NC	NC	NC	NC	NC	NC	NC	NC	NC	NC	NC	NC	NC	NC	NC	NC	NC	NC	NC	NC	NC	5	NC	NC	NC	NC	NC	NC
		
		**Hsa-no ortholog**																																		
		**Sce_Hpr1p**	NC	NC	NC	NC	NC	2	NC	NC	NC	NC	NC	NC	NC	NC	NC	NC	NC	NC	NC	NC	NC	NC	NC	NC	NC	NC	NC	NC	NC	NC	NC	NC	NC	NC
		
		**Hsa-no ortholog**																																		
		**Sce_Mft1p**	NC	NC	NC	NC	NC	2	NC	NC	NC	NC	NC	NC	NC	NC	NC	NC	NC	NC	NC	NC	NC	NC	NC	NC	NC	NC	NC	NC	NC	NC	NC	NC	NC	NC
**THO**																																				
		**Hsa-no ortholog**																																		
		**Sce_Thp2p**	NC	NC	NC	NC	NC	1	NC	NC	NC	NC	NC	NC	NC	NC	NC	NC	NC	NC	NC	NC	NC	NC	NC	NC	NC	NC	NC	NC	NC	NC	NC	NC	NC	NC
	
	
		**Hsa_UAP56**	1	1	1	1	1	1	1	1	1	2	1	1	1	1	1	1	1	1	1	4	1	1	1	1	1	1	1	1	1	1	1	1	2	1
		**Sce_Sub2**	1	1	1	1	1	1	1	1	1	2	1	1	1	1	1	1	1	1	1	4	1	1	1	1	1	1	1	1	1	1	1	1	2	1
		
	**TREX**	**Hsa-no ortholog**																																		
		**Sce_Yra1p**	NC	NC	3	2	NC	1	3	3	2	NC	NC	4	NC	4	2	2	NC	NC	NC	NC	NC	NC	NC	NC	NC	NC	NC	NC	3	NC	NC	NC	NC	4
		
		**Hsa_THOC3**	1	1	1	2	2	3	2	2	4	3	4	3	1	1	1	1	2	2	4	1	3	4	4	4	2	2	2	4	2	3	4	4	3	3
		**Sce_Tex1p**	3	5	3	3	3	1	NC	4	4	NC	3	5	3	3	3	3	4	NC	4	NC	3	NC	NC	NC	3	3	NC	NC	3	NC	NC	NC	NC	5

		**Hsa_TAP**	1	2	2	2	3	3	2	2	3	NC	4	4	NC	NC	NC	NC	NC	NC	NC	NC	NC	NC	NC	NC	NC	NC	NC	NC	NC	NC	NC	3	NC	NC
		**Sce_Mex67p**	3	3	3	NC	NC	1	2	3	3	NC	4	4	NC	NC	NC	NC	NC	NC	NC	NC	NC	NC	5	5	NC	NC	NC	NC	NC	5	5	4	NC	NC
**mRNA transport adaptor**																																				
		**Hsa_p15**	1	2	1	2	NC	3	1	4	2	NC	3	3	2	3	2	3	NC	NC	3	NC	3	NC	NC	3	NC	NC	NC	NC	3	2	2	3	NC	3
		**Sce_Mtr2p**	NC	NC	NC	NC	NC	1	NC	NC	NC	NC	NC	NC	NC	NC	NC	NC	NC	NC	NC	NC	NC	NC	NC	NC	NC	NC	NC	NC	NC	NC	NC	NC	NC	NC

		**Hsa_DDX19**	1	1	1	1	1	1	1	1	1	2	1	2	1	1	1	1	1	1	1	1	1	4	1	1	1	1	1	2	1	1	2	1	1	2
		**Sce_Dbp5p**	1	1	1	1	1	1	1	1	1	2	1	2	2	2	2	1	1	1	1	2	1	4	1	1	1	2	2	2	1	2	2	1	2	1
		
		**Hsa-no ortholog**																																		
		**Sce_Gle1p**	4	3	3	NC	NC	1	NC	2	3	NC	NC	NC	NC	3	NC	NC	5	NC	4	NC	NC	NC	NC	NC	NC	NC	NC	NC	NC	NC	NC	4	NC	NC
		
		
		**Hsa_RAE1**	1	1	1	1	2	2	2	1	1	2	1	3	1	1	1	1	1	1	2	2	2	4	2	2	4	2	1	1	1	3	3	2	3	3
		**Sce_Gle2p**	2	2	1	2	2	1	1	1	1	2	1	3	2	1	1	1	1	1	2	2	2	NC	2	1	4	2	1	1	1	3	2	2	3	2
**Additional export factors**																																				
		**Hsa_TPR**	1	5	3	4	NC	4	3	5	5	NC	4	NC	3	5	3	5	NC	4	NC	NC	NC	NC	NC	NC	NC	NC	3	3	3	NC	NC	5	NC	NC
		**Sce_Mlp1p**	2	4	3	NC	NC	1	2	3	NC	NC	NC	NC	4	NC	4	NC	NC	NC	NC	NC	NC	NC	NC	NC	NC	NC	5	4	5	NC	NC	5	NC	NC
		
		**Hsa-no ortholog**																																		
		**Sce_Nab2p**	4	NC	NC	NC	NC	1	3	4	3	NC	NC	NC	NC	NC	NC	NC	NC	NC	3	NC	NC	NC	4	5	NC	NC	NC	NC	NC	NC	NC	NC	NC	NC
		
		**Hsa_HNRNPM**	1	3	4	4	4	4	4	4	4	4	4	NC	4	4	4	4	4	4	4	4	4	4	4	4	NC	4	4	4	4	4	4	4	NC	4
		**Sce_Gbp2p**	4	2	4	4	3	2	3	3	4	4	4	4	4	4	4	4	4	4	4	3	4	4	4	4	4	4	4	4	3	4	2	4	4	4

Results were ranked into 5 categories (1-5; with 1 being the most conserved). NC (Not Classified). We present results for a representative subset of species in here, and full results are presented in Table S2 (Additional File 1)

### Ran-GTP dependent RNA export (Table 2; and Tables S2 and S3 from Additional File 1)

Ran is a small, soluble GTPase present in both the nucleus and cytoplasm of all eukaryotic cells that plays a critical role in RNA export. Exportins, the nuclear export transport receptors, bind nuclear cargo together with RanGTP in the nucleus to form a ternary complex (RNA-exportin-RanGTP) that is translocated to the cytoplasm, where the complex subsequently dissociates upon hydrolysis of RanGTP to release the cargo molecule. Nuclear export of miRNA, rRNA, snRNA, and tRNA has been shown to follow the RanGTP-exportin model. Bioinformatic searches showed that Ran was well conserved in all species included in this study, with a Ran CHS in category 1 (see categories definition in the Methods section) in almost all genomes surveyed, and high levels of sequence conservation. The Ran orthologs from human and *Trypanosoma brucei *are 72.2% identical/79% similar, whereas the *S. cerevisae *and *T. brucei *orthologs are 70.51% identical/79% similar. The high conservation scores and phyletic distribution of Ran CHS indicate that the LECA was highly likely to have possessed an ortholog of Ran, and extends previous work that included fewer eukaryotic species in their analyses [18,19].

Ran Binding Protein 1 (RanBP1, Yrb1p in yeast), another protein of the Ran cycle, had a high level of conservation as well. RanBP1 stimulates RanGTP hydrolysis by RanGAP [20,21]. Because we found strong CHS for RanBP1 in representatives of the three eukaryotic megagroups (Table 2), it is likely that RanBP1 was present in the LECA. Experimental evidence showing that RanBP1 is involved in the Ran cycle in the Excavate *Leishmania major *[22] would suggest its function has been conserved throughout eukaryotic evolution.

RCC1 (Srm1 in yeast) and RanGAP (Rna1 in yeast) are responsible for the establishment of a RanGTP-RanGDP gradient across the nuclear envelope that drives RanGTP dependent transport. These two proteins were less conserved than either Ran or RanBP1 in our searches (Table 2). RCC1 acts in the nucleus as the guanine nucleotide exchange factor, and RanGAP acts in the cytoplasm and regulates the GTPase activity of Ran. We only found CHS for the human or yeast RCC1 and RanGAP orthologs in categories 1 and 2 in opisthokonts (Table 2). Because we found some CHS in category 3 for RCC1 and RanGAP among plants, chromalveolates, and excavates, we infer that these two proteins were probably present in the LECA (Table 2).

The proteins involved in the Ran cycle discussed above play role in intracellular transport of proteins and nucleic acids. Their strong conservation across all species examined is in line with previous reports [18-23]. Further, these results are consistent with the predictions of the evolutionary scenario presented by Jékely [24], where RanGTP-dependent transport is strongly related to the origin of the nucleus. In addition to the proteins involved in the Ran cycle, other export factors are required to provide specificity for the different RNA export mechanisms. In the following sections we discuss conservation for the proteins that are specifically involved in the different RNA export pathways.

#### MicroRNA and tRNA export pathways

MicroRNAs are short non-coding RNAs involved in the regulation of gene expression in eukaryotic cells [25], and tRNAs are non-coding RNAs that transfer specific amino acids to a growing polypeptide chain. In the case of miRNA and tRNA export, the exportins involved in the export of miRNA precursors in human and yeast is exportin-5 (XPO5, Msn5 in yeast), whereas XPOt (Los1 in yeast) is the one involved in tRNA export [26].

There were no conservation scores better than 3 for yeast XPO5 other than its CHS in yeast (Table 2). Conversely, conservation scores outside members of Metazoa were 3 or worse in searches involving human XPO5. These results agree with previous studies that had failed to find XPO5 homologs in members of Euglenozoa and Apicomplexa [27,28]. Interestingly, several of the species that lack XPO5 homologs, such as *T. brucei *or *Trichomonas vaginalis*, posses a functional miRNA machinery [29,30], suggesting that there are additional pathways for the export of miRNA other than the one used in humans. A simple explanation for the complex phyletic distribution of XPO5 CHS is difficult to reconcile with current eukaryotic phylogenies. There are known orthologs of XPO5 in Plantae, and Opisthokonta, and there are CHS of score 3 in the three different megagroups, which would suggest that an XPO5 ortholog was probably present in the LECA and would have been secondarily lost in several lineages. Under alternative eukaryotic phylogenies [9-14] the presence of orthologs in plants and opisthokonts would suggest that XPO5 was probably present in the LECA, as previously suggested [28].

In the case of XPOt, CHS for the yeast ortholog in category 1 were only found in Fungi, whereas CHS for the human ortholog in categories 1 and 2 were found among members of Metazoa and Amoebozoa, respectively (Table 2). In addition, we found CHS in category 3 or higher for the human XPOt ortholog in members of Opisthokonta, Plantae, Chromalveolata and Excavata. Although some of the CHS in *Leishmania *and *Trypanosoma *(Excavata) and *Plasmodium *(Chromalveolata) are not strongly conserved, phylogenies show that they are true orthologs [28]. Orthologs of XPOt are involved in tRNA export in yeast and plants, but they are not an essential factor in some species. For example, nucleocytoplasmic export of tRNA is not fully blocked when the XPOt gene is deleted in *S. cerevisae*, *S. pombe *and *A. thaliana *[31-33]. Similar to XPO5, orthologs of XPOt are present in most of the major eukaryotic supergroups in this study, which would imply that the LECA was likely to posses an ortholog of this gene.

XPO5 has also been postulated to play a minor role in tRNA export for some organisms. In this case, XPO5 mediates the tRNA transport in association to a ribosomal elongation factor named eEF1A, TEF2 in yeast [34,35]. This ribosomal elongation factor is also highly conserved, with CHS in category 1 for most species in this study (Table 2), once again, suggesting that it was likely present in the LECA.

#### Small nuclear RNAs export pathway

Small nuclear RNAs are a group of non-coding transcripts involved in RNA splicing, transcription factor regulation and telomere maintenance. The different snRNAs are synthesized in the nucleus, assembled into snRNPs in the cytoplasm and re-imported into the nucleus. Export of snRNAs presents some departures from the simpler model of exportin-RanGTP-cargo seen in miRNAs and tRNAs. In this case the exportin does not bind RNA directly, and additional adaptor proteins are needed. Exportin-1 (XPO1, CRM1 in yeast) is the transport receptor in charge of carrying snRNAs to the cytoplasm, in association to CBP20 (CBC2 in yeast), which binds to the snRNA; CBP80 (Sto1 in yeast), which ensures high affinity binding; and PHAX, which provides the nuclear export signal [36,37].

Our analyses indicate that XPO1 is the most conserved exportin among the three included in our study (Table 2). There were CHS for human and yeast orthologs in categories 1 and 2 in most of eukaryotic lineages, and sequence conservation was also high. The human and *T. brucei *XPO1 orthologs were 32% identical/52% similar. In the case of CBP20 we found CHS in categories 1 or 2 in all eukaryotic supergroups, whereas we failed to find CHS of CBP80 in several species (Table 2). By contrast, conservation scores for PHAX are either low, or below our detection threshold for all eukaryotes in this study other than members of Metazoa, in agreement with a previous study [36]. These results indicate that the LECA was likely to have XPO1 and CBP20 orthologs, but that PHAX appears to be a lineage-specific innovation. The case for CBP80 is more complex, there are some matches in category 3 in all eukaryotic megagroups, but no CHS were identified in several species. We infer that CBP80 could have been present in the LECA, but that it was either lost or diverged beyond our ability to detect it in several species.

#### rRNA export pathway

Ribosomes are made up of a combination of different rRNAs and a large number of ribosomal proteins that are organized into the large (60S) and small (40S) pre-ribosomal subunits. In yeast, nuclear export of the pre-ribosomal subunits depends on XPO1 and RanGTP [38,39], and also involves additional proteins. In yeast, the nuclear export of the pre-60S ribosomal subunit requires the proteins Nmd3, Arx1 and the Mex67/Mtr2 heterodimer, but export of 40S is poorly understood [40-42]. The role of Nmd3, Arx1 and the Mtr2/Mex67 has not been completely elucidated yet. On the one hand, deletion of the Arx1 gene leads to pre-60S accumulation in the nucleus [43]. On the other hand, a subsequent study from the same group shows that in the absence of Arx1, the addition of a nuclear export signal to the pre-60S subunit would be enough to restore its export [44], which suggests that the export of pre-60S is relatively flexible.

XPO1 conservation scores have already been discussed with the other snRNA export proteins. Results for the two subunits of the general transport adapter, (TAP/p15 in human, Mex67/Mtr2 in yeast) are low (Table 3) and will be discussed in the next section because of their major role in mRNA export. The phyletic distribution of XPO1, XPO5, and XPO5 suggests that these exportins are involved with RNA export in all the different eukaryotic supergroups analyzed, and also indicate that each of them is traceable to the LECA. We observed that XPO1, which is mostly involved in rRNA export in opisthokonts, is the most conserved one, while XPOt and XPO5, involved in miRNA, tRNA, snRNA export, are not as conserved. Even though orthologs of these exportins are found among excavates and chromalveolates, their role in these lineages is not fully understood. The little functional data available suggest that their roles are not entirely the same as in opisthokonts. For example, XPO1 has been shown to be involved with mRNA export in the parasites of the genus *Trypanosoma *[45], but it is mostly involved with rRNA export in opisthokonts. Based on the functional data and their phyletic distribution, we speculate that there is some level redundancy in the functional role of the different exportins and propose that in the LECA, XPO1 could have acted as the protein responsible for transport of most of the RNAs through the NPC.

For the additional rRNA export proteins, our analyses show that Nmd3 is well conserved in the vast majority of species included in this study (Table 2), with 58% similarity/38% identity in comparisons between human and *T. brucei *orthologs. This would indicate that Nmd3 is highly likely to have been present in the LECA. By contrast some of the additional adaptors, such as Arx1, Alb1p, and PHAX have few matches in category 3, and seem to be lineage specific innovations. Strong CHS for Arx1 are only found among Fungi, strong CHS for Alb1p are restricted to yeasts in the subphylum Saccharomycotina, whereas strong CHS for PHAX are only found among animals.

As in the case with the exportins, the phyletic distribution of the transport adaptors discussed above suggests that these proteins are involved with export of tRNA, miRNAs, snRNAs and rRNAs in all the different eukaryotic supergroups, and that they are traceable to the LECA, such as Nmd3, which also appear to be conserved among the different eukaryotic supergroups. The specific combination of exportins and adaptors varies in a lineage-specific manner through a combination of gene gains and losses. For example, XPO5 has been secondarily lost in fruit fly, whereas XPOt has been secondarily lost in *C. elegans. *On the other hand, we also found that Arx1, Alb1p and PHAX are restricted to different groups within Opisthokonta, suggesting that they are innovations specific to this group. These results would suggest that the specific export mechanism for the different RNAs varies in a lineage-specific manner.

### RanGTP-independent transport: mRNA export pathway (Table 3; and Tables S2 and S3 from Additional File 1)

In Metazoa and Fungi, nucleocytoplasmic export of the majority of the mRNAs does not follow the RanGTP-dependent exportin pathway. From yeast to humans, mRNAs associate with protein factors into messenger RNPs (mRNPs) which are then exported through the NPC by an essential shuttling heterodimer, TAP/p15 in human and Mex67/Mtr2 in yeast, which functions as a general mRNA export receptor to transport mRNPs through the NPCs [46-48]. The dimeric export receptor operates in association with TREX (Transcription/Export), a multiprotein complex that connects transcription with mRNA export [49,50]. Human TREX consists of the RNA helicase UAP56 (Sub2 in yeast), the RNA-binding adaptor protein Aly (Yra1 in yeast) and the THO complex [51-53]. In yeast, an additional complex named TREX-2, consisting of Thp1, Sac3, Sus1, and Cdc31 is also capable of mediating the nuclear export of mRNPs in concert with Mex67/Mtr2 [54-56].

Our analyses indicate that few of the proteins involved in the mRNA export pathway are conserved across all eukaryotes. This is perhaps not unexpected given the differences in mRNA processing among eukaryotes. In the case of the TAP or Mex67 subunit of the heterodimer, conservation scores outside Fungi and Metazoa were 4 or 5 with the exception of some Excavata members. Whereas bioinformatic searches for the yeast Mtr2 subunit only identified strong CHS in fungi. This is not a surprising result, as similarities between Mtr2 and its human functional analog, p15, are restricted to three-dimensional structure [57], which cannot be detected by sequence similarity analyses. In the case of TREX, the UAP56 subunit (Table 3) is well conserved across all eukaryotes: we have strong CHS for the yeast ortholog in all species in this study except for *Emiliana huxleyi*. The Thoc3 subunit, Tex1 in yeast (Table 3), has good CHS among opisthokonts and plants, and moderate candidates in Amebozoa, Chromalveolata, and Excavata. By contrast, we could not identify CHS for the yeast Yra1 (Table 3) in several Chromalveolata and Excavata. A similar situation is observed for the TREX-2 complex, where only CETN3, CDC31 in yeast (Table 3), is well conserved across all eukaryotes. CETN3 is a centrin involved in the duplication and segregation of the centrosome during cytokinesis that is also involved in mRNA export in yeast [55]. However, evidence for a role of CETN3 in mRNA export in some members of Excavata is lacking, as it is only required for the initiation of cytokinesis in *Leishmania *and *T. brucei *[58].

The cellular fate of the different mRNAs depends mainly on the shuttle proteins TAP/p15, Yra1, Rae1 and Gle1, DDX19, which are also required for proper translation [4,59]. Rae1 acts in delivering TAP to the NPC [60] and DDX19 is responsible for triggering the mRNPs remodeling at the NPC cytoplasmic-face scaffold, delivering the mRNA to the cytoplasm [61]. Both Rae1 and DDX19 are well conserved, with CHS in categories 1 and 2 for all the supergroups. However, the adaptor protein Gle1, that plays an important role in the control of mRNA export in yeast [62], appears to be missing from Amoebozoa, Plantae, Chromalveolata, and Excavata, suggesting it is an innovation specific to Opisthokonta.

The proteins involved in the RanGTP-independent export of mRNA are generally less conserved than those involved in RanGTP-dependent export of all other RNA species. Some of the major components of the mRNA export pathway are conserved across the different eukaryotic supergroups examined. This is the case for UAP56, DDX19, and Rae1. However, there are many proteins that play essential roles in mRNA export in opisthokonts that lack strong CHS outside this group, and appear to represent opisthokont-specific innovations. This group includes Mlp1, Mlp2, and the shuttling proteins TAP, Yra1, Nab2, and Gbp2. These results suggest that mRNA export in members of the other supergroups might follow slightly different mechanisms. One possibility is that mRNA export might also follow a RanGTP-dependent pathway. Interestingly, the fact that in the excavate *Trypanosoma *the XPO1 ortholog is involved in mRNA export [45] is consistent with our inference. We speculate that mRNA might have been exported by XPO1 in a RanGTP-dependent manner early in eukaryote evolution, and that it later followed more specialized pathways.

## Conclusions

Results from our genomic comparisons indicate that several of the key proteins involved in the different RanGTP-dependent RNA export pathways are conserved across most eukaryotic lineages, and thus we infer that orthologs of them were highly likely to have been already present in the LECA. Examples of these are the exportins XPO1 and XPOt, Nmd3 and most of the proteins involved in the Ran cycle. Despite the relatively strong level of conservation, we also document how these export mechanisms vary in a lineage-specific manner as a consequence of the differential gene gains and losses, as documented by the secondary loss of XPOt in *C. elegans*, and the emergence of Arx1 in Fungi and of PHAX in animals. In agreement with inferences drawn from studies of components of the cytoskeleton, endomembrane, NPC, and spliceosome [17-19,62-67] a number of key RNA nucleocytoplasmic transport factors can be traced to the LECA. This would suggest that the different RanGTP-dependent RNA export mechanisms were already present in the LECA.

By contrast, our analyses indicate that several of the proteins involved in the RanGTP-independent export pathway from opisthokonts are lineage-specific innovations. The mRNA export pathway is the most complex and the least conserved among those examined in this study. We found CHS for few of the yeast proteins involved in mRNA export in most eukaryotic lineages, which suggests that mRNA export among them is different from what is observed in yeast. We document the acquisition of several lineage-specific innovations in the mRNA export mechanisms of opisthokonts relative to the other supergroups included in this study. It seems plausible that the evolution of a RanGTP-independent mRNA export pathway in opisthokonts might be related to the observed differences in the regulation of gene expression. In Amoebozoa, Chromalveolata and Excavata, most regulation is post-transcriptional [68], whereas in human and yeast the presence of both transcriptional and post-transcriptional regulation is linked to a more refined control of gene expression. One possibility is that having an mRNA export pathway fully separated from the other RNA export pathways increases of the range possibilities of gene-specific control. This would be particularly important to allow both coordination and versatility in gene expression control [69,70], as well as to open additional opportunities to connect active transcriptional sites to the NPC, allowing a fine control of gene expression in yeast and human [71,72]. Functional data regarding mRNA export in excavates and chromalveolates is still limited, and despite the fact that some studies suggest if follows less complex routes [45], the possibility that alternative specializations haven arisen in these groups cannot be discarded.

**Figure 4:**
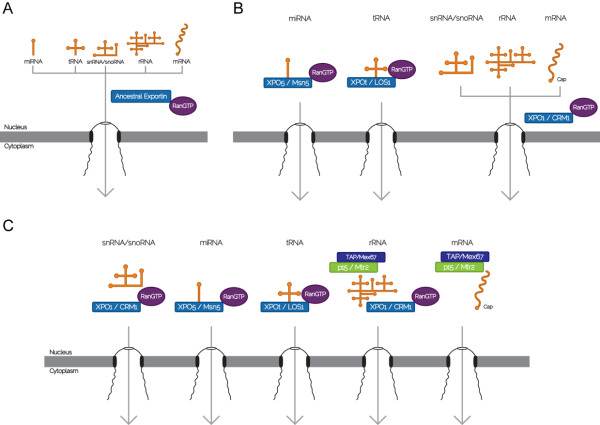
**Model proposed of the evolution of the different RNA export pathways throughout eukaryotic history**. (A) Early in the history of eukaryotes a single ancestral exportin similar to XPO1 was in charge of nucleocytoplasmic export of all RNAs in a RanGTP-dependent manner. (B) Prior to the emergence of the LECA, different exportins originated and specialized in nucleocytoplasmic export of the different RNA species. (C) In opisthokonts, a further innovation was the emergence of a RanGTP-independent pathway of mRNA export. This pathway relies on the TAP-p15 heterodimer as the transport receptor instead of an exportin, and includes several other adaptors which are restricted to opisthokonts. Each box represents a human/yeast protein.

Taken together, our analyses suggest that the LECA possessed most of the basic components of the different RanGTP-dependent RNA export mechanisms, which are also well conserved among the different eukaryote supergroups included in this study. In addition, we also show that some of the major components of the RanGTP-independent mRNA export pathways can also be traced to the LECA, but that several of the proteins that play key roles in opisthokonts derive from lineage-specific innovations. It is likely that early in eukaryote evolution a single generalized ancestral exportin was probably responsible for nucleocytoplasmic transport of all RNA species (Figure 4A). Prior to the emergence of the LECA, orthologs of XPO1, XPO5, and XPOt would have already emerged and specialized in transporting a subset of the RNAs (Figure 4B). We speculate that in the LECA mRNA might have been exported by XPO1 in RanGTP-dependent manner, as in excavates in the genus *Trypanosoma*. The emergence of the RanGTP-independent mRNA export pathway in opisthokonts coincides with the origin of several lineage-specific innovations, and might be related to refinements in the regulation of gene expression in this supergroup (Figure 4C).

## Authors' contributions

MS, NMV, ARA and FGH designed the research. MS and ARA identified the yeast and human queries. NMV and MS did the bionformatic searches. MS, NMV, SG, ARA and FGH discussed the results. MS, ARA and FGH wrote the manuscript. All authors read and approved the final manuscript.

## Supplementary Material

Additional File 1 **Spreadsheed file containing tables S1-S5**. **Table S1**. Full list of human and yeast proteins used as queries. ^a^- yeast orthologs in human, according to Ensembl. ^b^- Not described as a yeast ortholog in Ensembl, used as negative control. **Table S2**. Results for all proteins in all species with BLASTP (BLOSUM62) and PSI-BLAST (2 iterations) ranked in 1-5 code according to Methods. Code: Category of similarity, from 1(most conserved) to 5. RBH: Reciprocal Best Hit, 1:True; 0: False. NC: Not Classified. NA: Not Analyzed. **Table S3**. Individual results for each protein, using different algorithms for ranking 1-5 code. I: %Identity, S: %Similarity. QC: Query Coverage. **Table S4**. Comparative analysis of U5snRNP spliceosomal complex using 1-5 ranking according to Methods in all species. **Table S5**. Comparison of the protein repertoire in relation to further evolutionary studies. In blue, common proteins analyzed in at least one study. In red, proteins that were not analyzed in previous works. Click here for file

Additional File 2 **Exemplary alignments of proteins from the different categories**. Click here for file
