# Supplementary material for: Comparative genomics of proteins involved in RNA nucleocytoplasmic export
Source: BMC Evol Biol. 2011 Jan 11;11:7. doi: 10.1186/1471-2148-11-7 (PMC3032688; doi:10.1186/1471-2148-11-7)

Categories	Criteria
1	S ≥ 60%, HSP/QC ≥ 80%, QL ~ SL
2	S ≥ 50%, HSP/QC ≥ 60%, QL ~ SL
3	S ≥ 40%, HSP/QC ≥ 45%, QL ~ SL
4	S ≥ 30%, HSP/QC ≥ 30%
5	S ≥ 30% or HSP/QC ≥ 30%
NC	E-value > E-05

Figure S1. Categories are in 5-color degree-scale, matching the 1-5 similarity categories, with the respective criteria, defined in Methods. S = Similarity, HSP = High-scoring Segment Pair, QC = Query Coverage, QL = Query Lenght, SL = Subject Length, ~ = similar length.

Figure S2. Examples of alignments for human Exportin-1 (query Hsa_XPO1) in different eukaryotes (A-D). A: Category 1, B: Category 2, C: Category 3, D: Category 4, E: Category 5.

A

Hsa_XPO1 x Ngr (*Naegleria gruberi*)

S = 68.7%, QL = 1071, SL = 1064, HSP/QC = 98% E-value 0.0

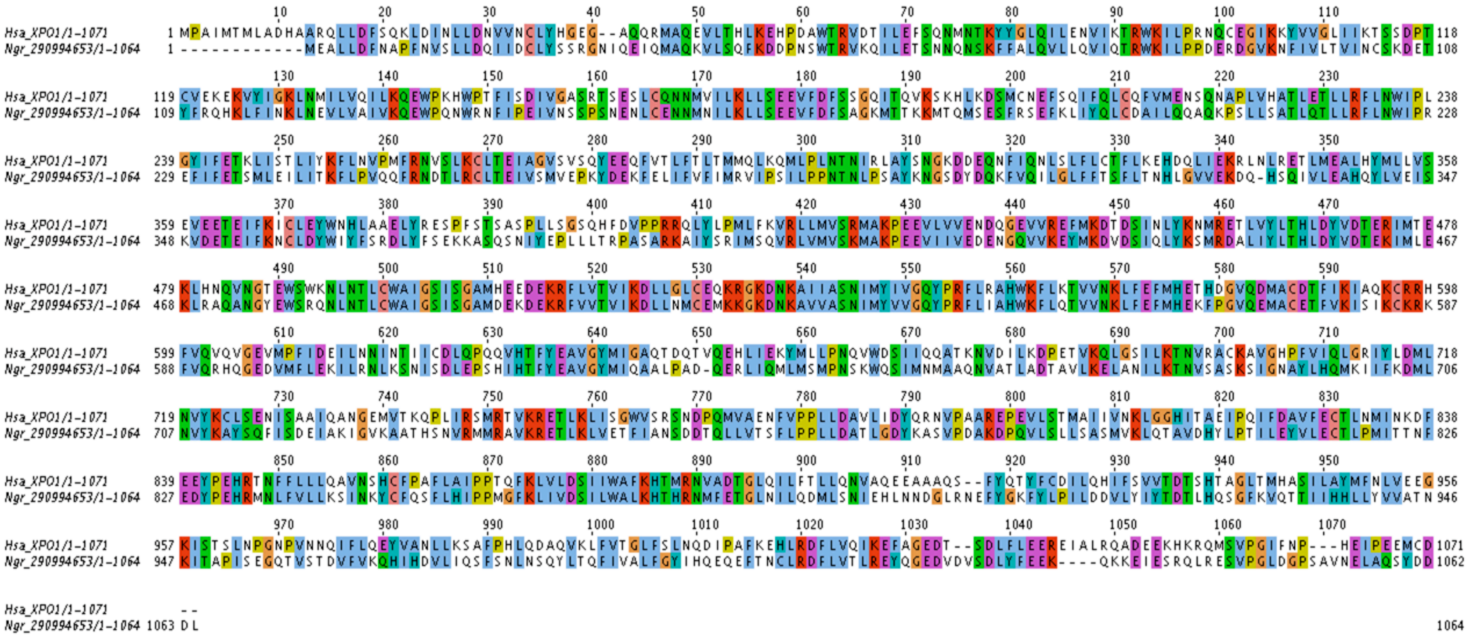

B

Hsa_XPO1 x Pfa (*Plasmodium falciparum*)

S = 56.3%, QL = 1071, SL = 1254, HSP/QC = 96%, E-value 2.21 E-82

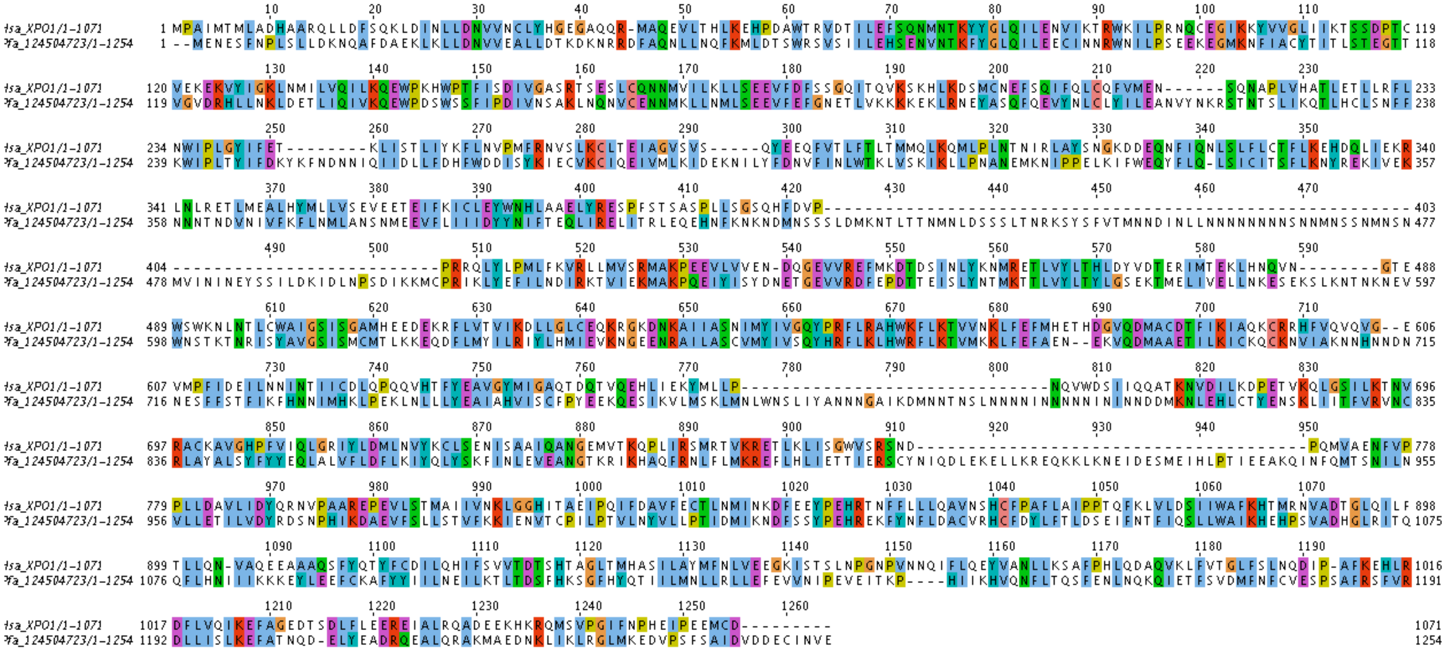

C

Hsa_XPO1 x Tva (*Trichomonas vaginalis*)

S = 46.9%, QL = 1071, SL = 1003, HSP/QC = 95%, E-value 2.03 E-70

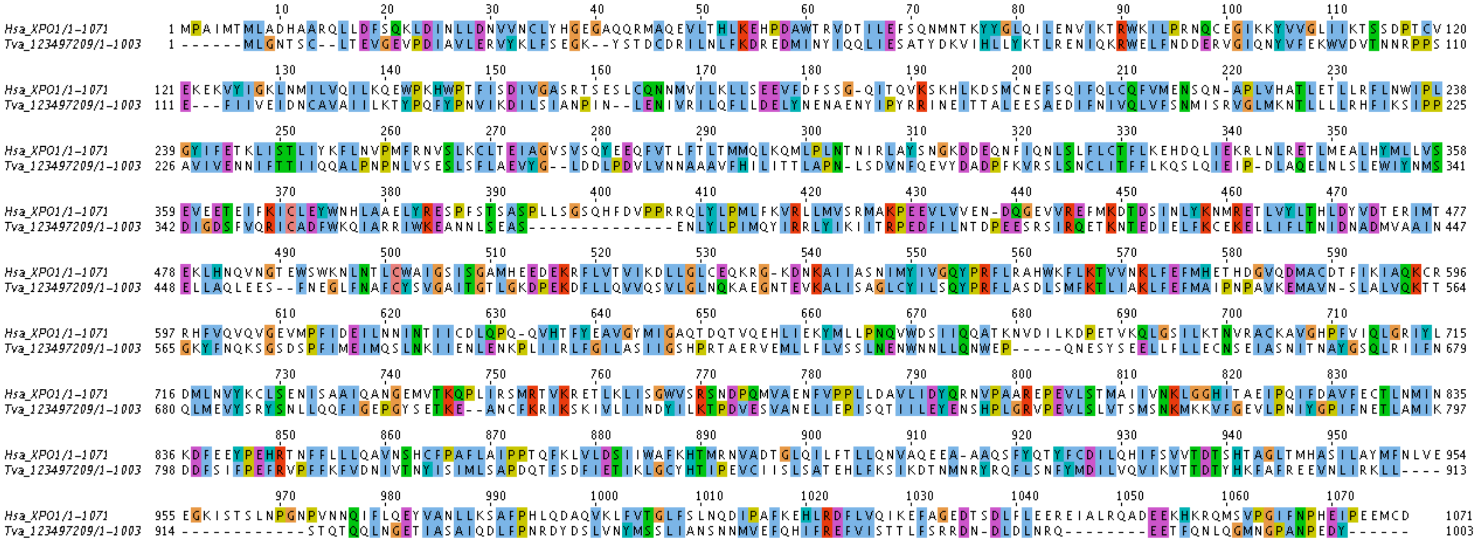

D

Hsa_XPO1 x Pte (*Paramecium tetraurelia*)

S = 60.1%, QL = 1071, SL = 572, HSP/QC = 51%, E-value 1.96 E-102

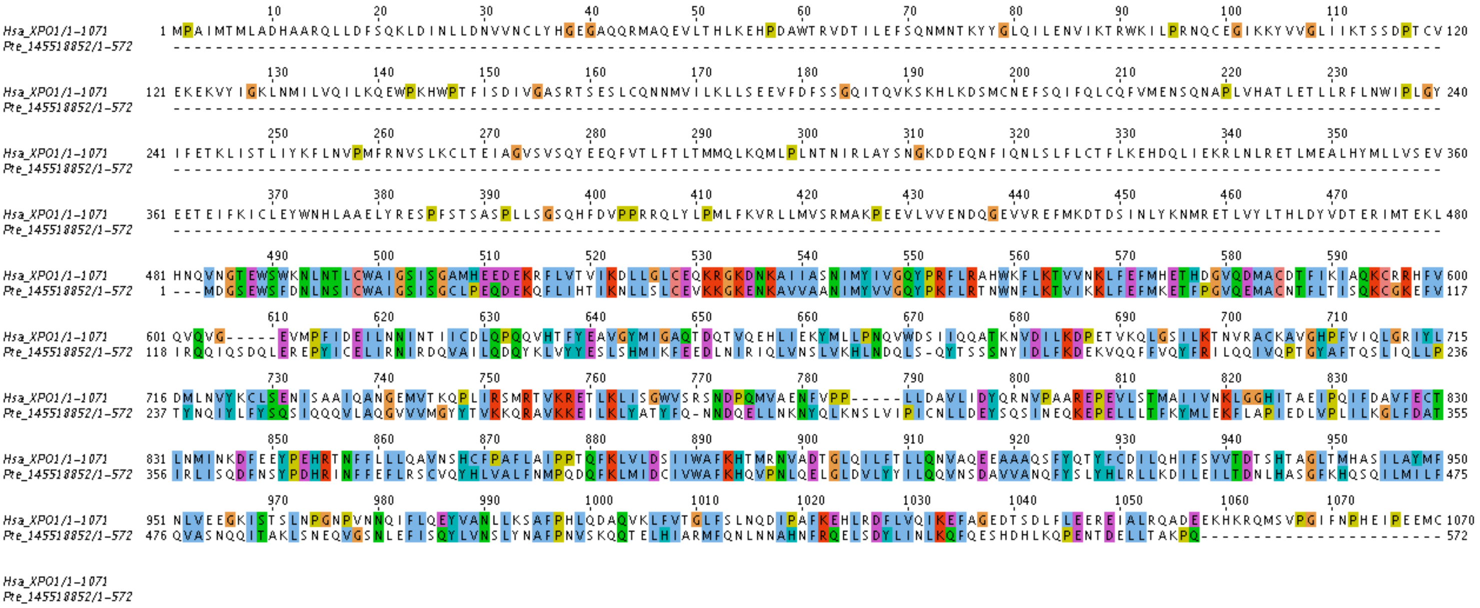

E

Hsa_XPO1 x Pbe (*Plasmodium berghei*)

S = 64.1%, QL = 1071, SL = 169, HSP/QC = 14%, E-value 1.58 E-29

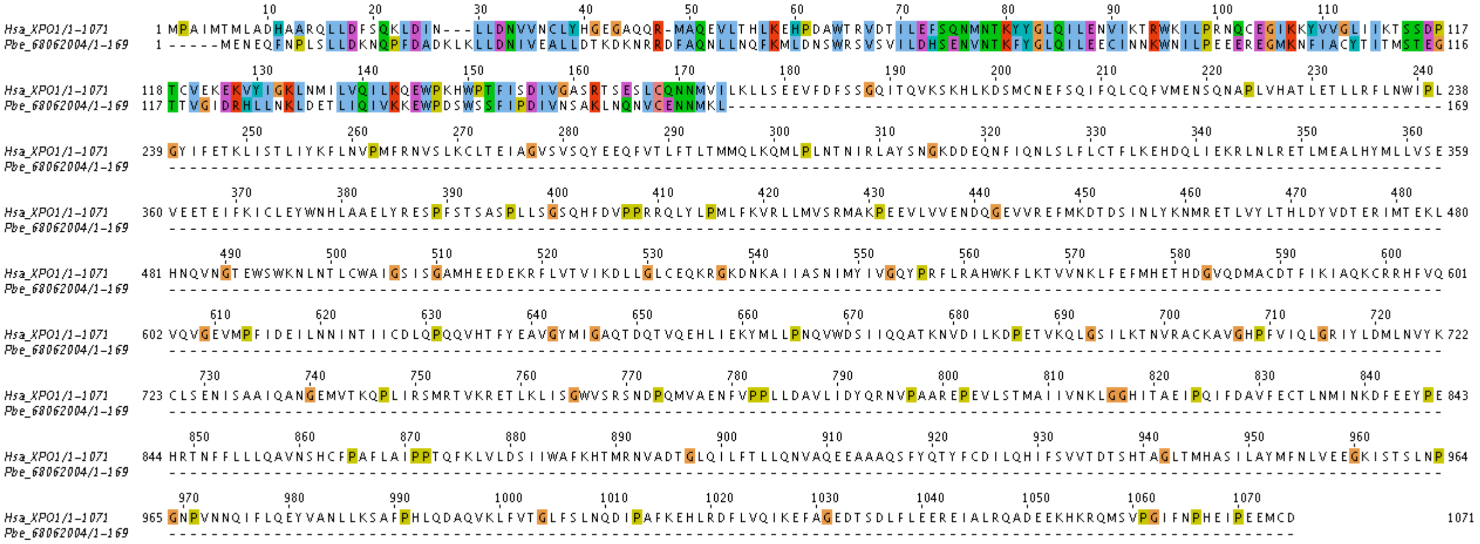

Figure S3. Examples of multiple alignments for human Rae1 (query Hsa_Rae1) in different eukaryotes (A-D). A: Category 1, B: Category 2, C: Category 3, D: Category 4, E: Category 5.

A

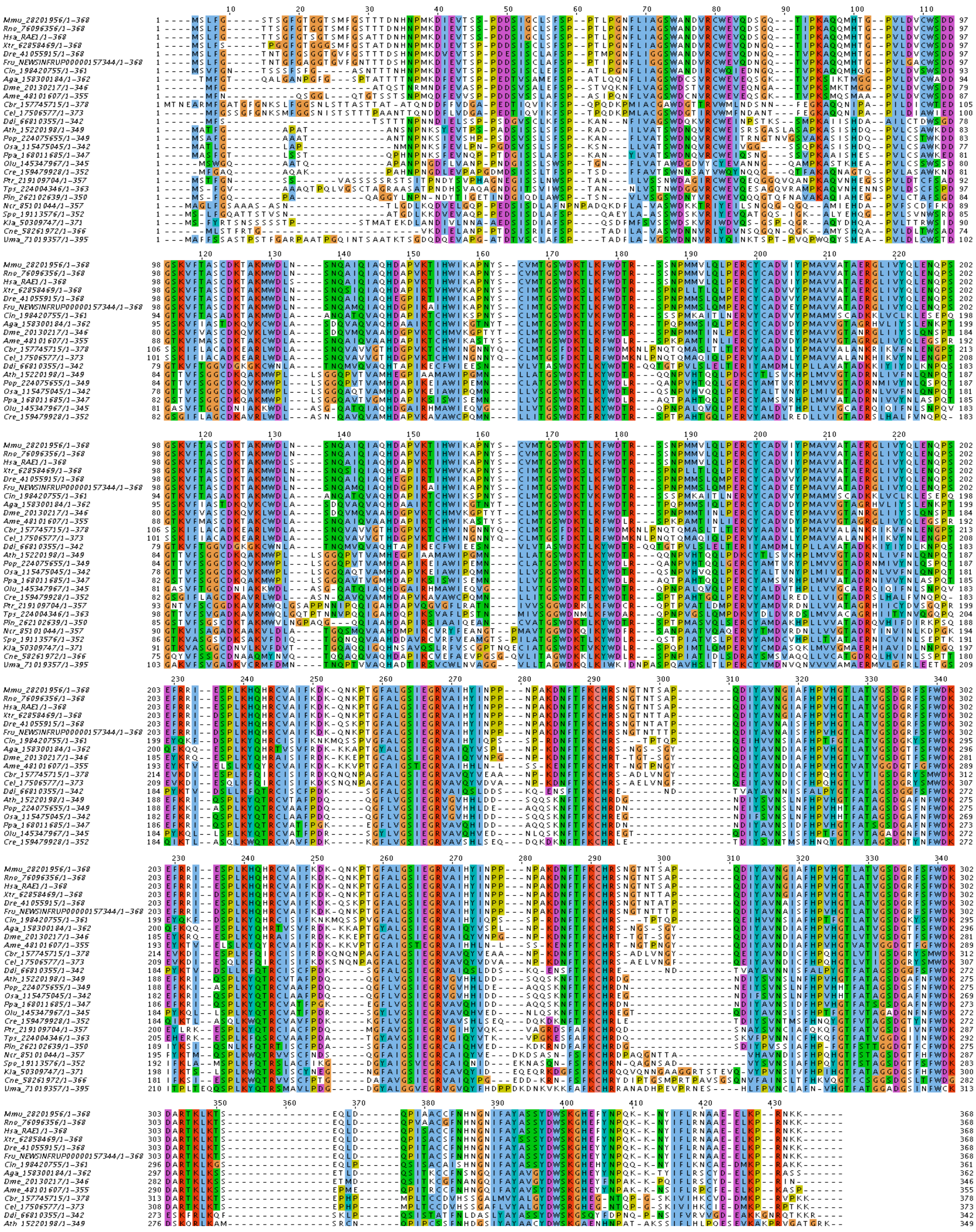

B

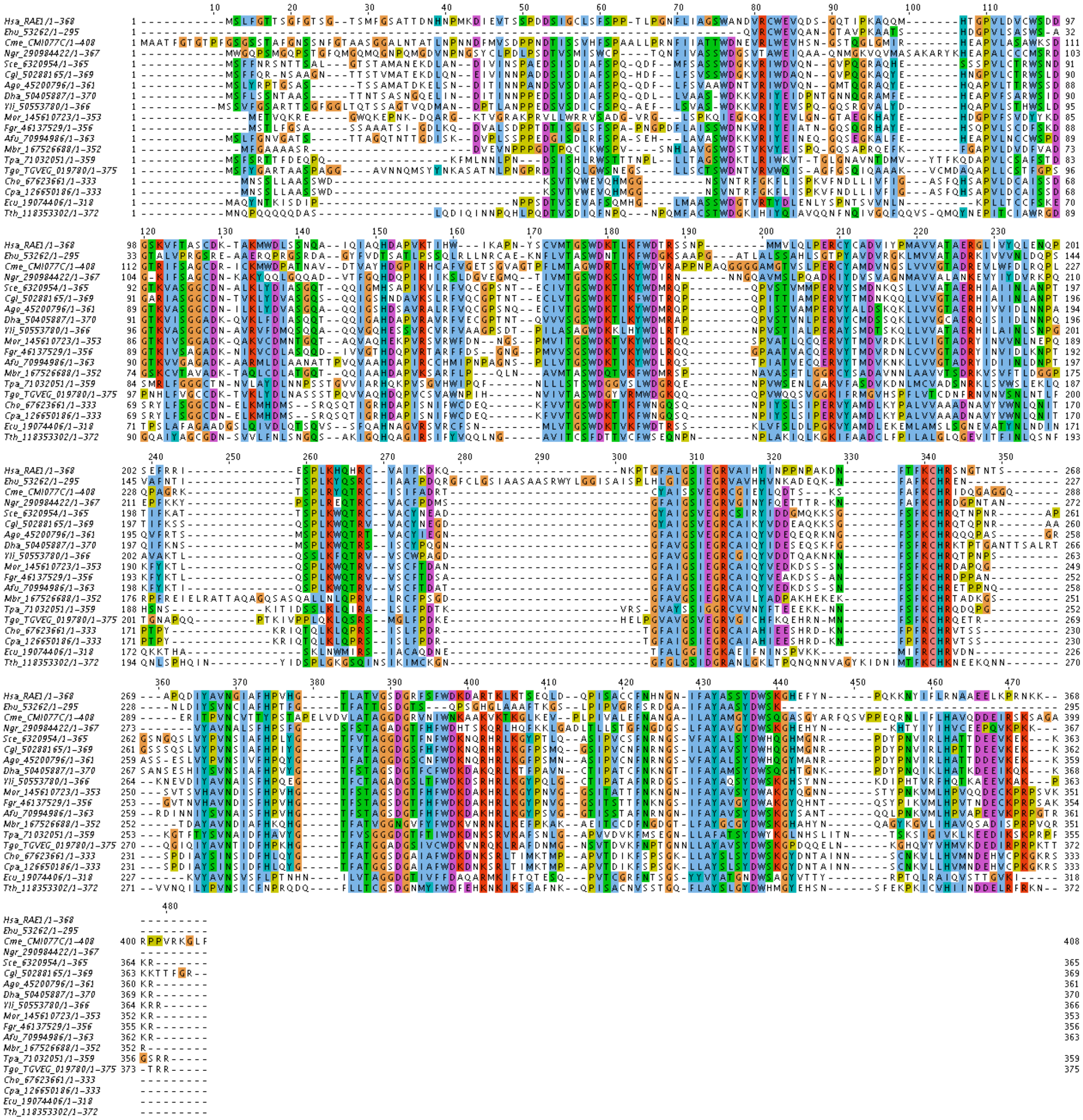

C

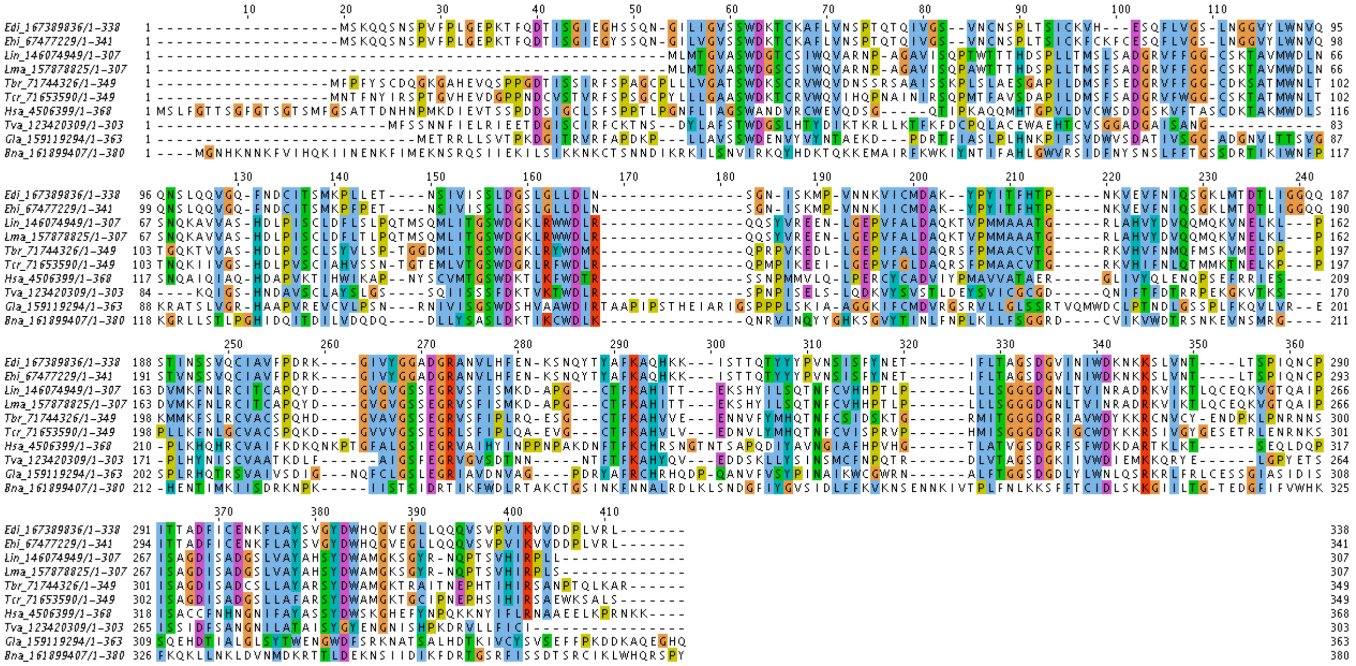

Edi_167389836/1-338
Ehi_67477229/1-341
Lin_146074949/1-307
Lma_157878825/1-307
Tbr_71744326/1-349
Tcr_71653590/1-349
Hsa_4506399/1-368
Tva_123420309/1-303
Gla_159119294/1-363
Bna_161899407/1-380

D

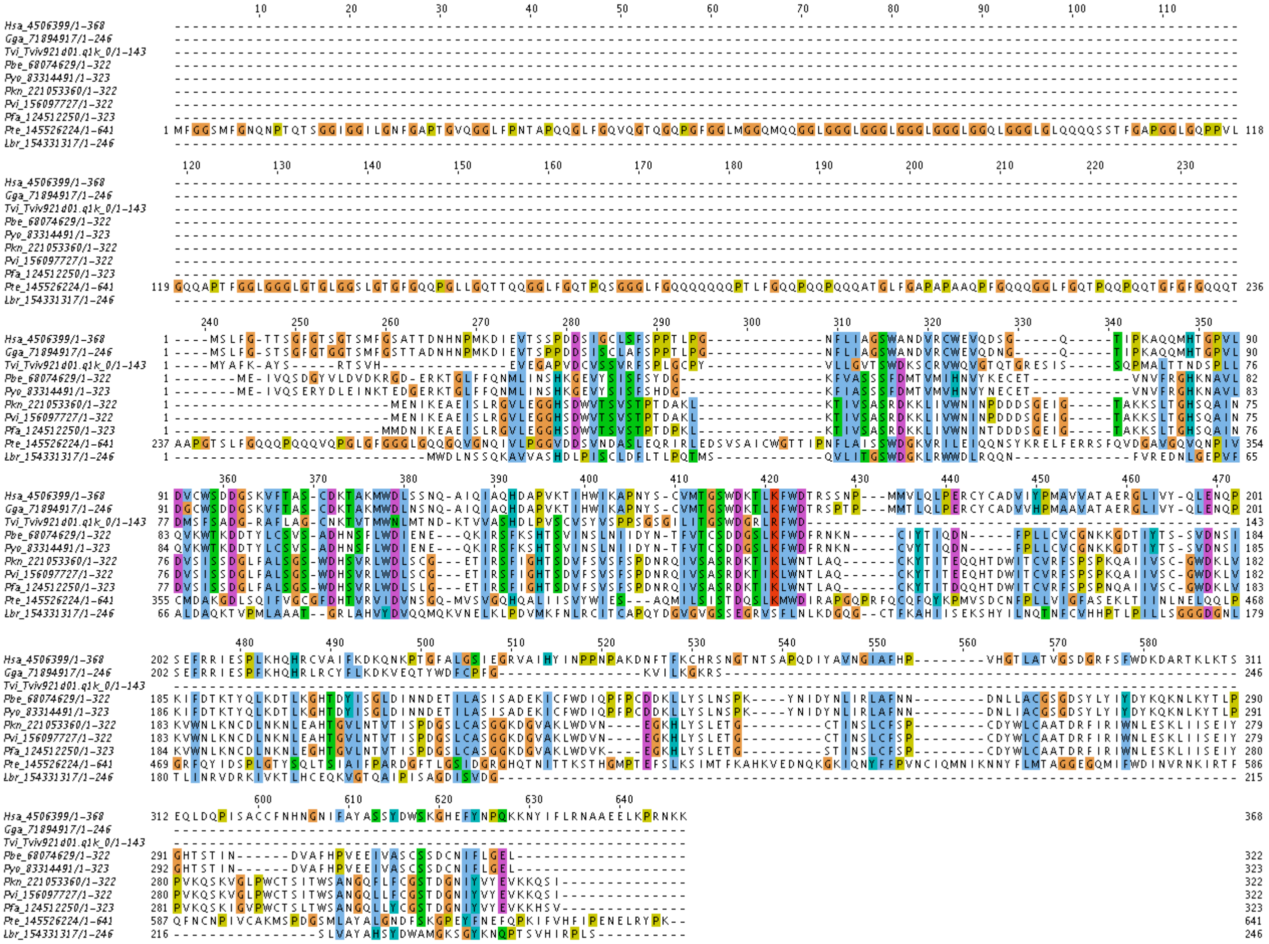

Hsa_4506399/1-368
Gga_71894917/1-246
Tvi_Tviv921d01.q1k_0/1-143
Pbe_68074629/1-322
Pyo_83314491/1-323
Pkn_221053360/1-322
Pvi_156097727/1-322
Pfa_124512250/1-323
Pte_145526224/1-641
Lbr_154331317/1-246

E

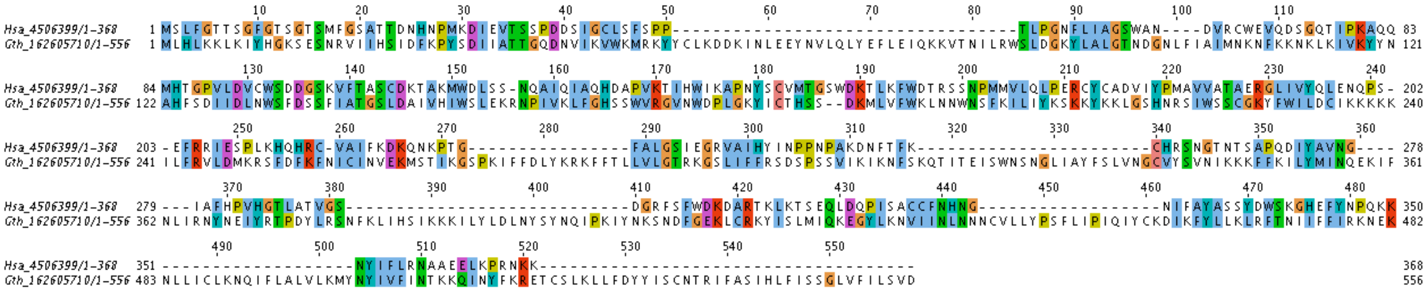

Supplement: Additional File 2 — Exemplary alignments of proteins from the different categories. [file 1471-2148-11-7-S2.PDF]
